# The association between sarcopenia and functional disability in older adults

**DOI:** 10.1016/j.jnha.2023.100016

**Published:** 2024-01-01

**Authors:** Hui Zhou, Xiong Ding, Meijie Luo

**Affiliations:** a Nursing Department, The Third Xiangya Hospital, Central South University, Changsha, Hunan, China; b Xiangya School of Nursing, Central South University, Changsha, Hunan, China; c School of Public Health, Wuhan University, Wuhan, Hubei, China; d Jinhua Polytechnic, Jinhua, Zhejiang, China

**Keywords:** Sarcopenia, Functional disability, Older people, Activities of daily living

## Abstract

**Objectives:**

Sarcopenia is associated with functional disability in older adults. However, no consistent conclusions have been reached considering the differences in the measurement and criteria of sarcopenia. We aimed to examine the association between sarcopenia status and functional disability based on China Health and Retirement Longitudinal Study (CHARLS).

**Design:**

A nationally representative longitudinal study.

**Setting and participants:**

Participants aged at least 60 years old from the CHARLS 2015y were included.

**Measurements:**

Sarcopenia was assessed according to the Asian Working Group for Sarcopenia 2019 algorithm. The outcomes of this study were basic activities of daily living (ADLs) and instrumental activities of daily living (IADLs). The logistic regression model was conducted to analyze the cross-sectional and longitudinal associations between sarcopenia status and ADLs and IADLs disability.

**Results:**

In the cross-sectional study, 37.2% of the 6893 participants were defined as having sarcopenia. Any form of sarcopenia was associated with ADLs and IADLs disability. During three years of follow-up, 786 (16.5%) participants developed new‐onset ADLs disability, and 980 (22.5%) participants developed new‐onset IADLs disability. Compared with the no‐sarcopenia, participants with possible sarcopenia (OR: 1.62, 95%CI: 1.34−1.95), sarcopenia (OR: 1.58, 95%CI: 1.18−2.11), or total sarcopenia (OR: 1.58, 95%CI: 1.34−1.88) had a higher risk of ADLs disability. While, the risk of IADLs disability for participants with possible sarcopenia (OR: 1.68, 95%CI: 1.41−2.00), sarcopenia (OR: 1.87, 95%CI: 1.40−2.51), or total sarcopenia (OR: 1.71, 95%CI: 1.45−2.00) was still significantly increased. With statistical interaction between sarcopenia status and residence or sex in ADLs and IADLs disability, older adults in urban, with ORs ranging from 2.14 to 2.44, were at a higher risk of functional disability than those in rural. Possible sarcopenia was associated with a much higher risk of ADLs disability (OR: 1.68, 95%CI: 1.26–2.25) in males and a higher risk of IADLs disability (OR: 1.98, 95%CI: 1.56–2.52) in females.

**Conclusions:**

Sarcopenia was associated with an increased risk of ADLs and IADLs disability among older Chinese adults. Even possible sarcopenia still significantly impacted ADLs and IADLs disability, and this association varied by sex and residence.

## 1 Introduction

As the aging process deepens, national and international health policies around the globe are increasingly focused on promoting health and preventing the disability of older adults for as long as possible [1]. The most common measures of functional disability are basic activities of daily living (ADLs) and instrumental activities of daily living (IADLs) [2]. In six low- and middle-income countries, the prevalence of functional disability among older adults ranged from 16.2% in China to 55.7% in India [3], causing great disease burden and medical care needs [4,5]. Skeletal muscle function is a core element for maintaining independence [6]. Sarcopenia is a skeletal muscle disease characterized by impairment of muscle strength and function due to progressive loss of muscle mass [7], and its components, like low muscle mass, muscle strength, and physical performance, were all negatively correlated with functional disability [8]. However, scarce evidence comes from developing countries, especially China, which has the world’s largest old population.

Sarcopenia is prevalent among older adults, and the prevalence ranges from 10% to 27% [9] and varies by diagnostic criteria and region. Several studies proved that sarcopenia was a risk factor for functional disability, with the odds of disability two times greater in individuals with sarcopenia than those without [10,11]. Epidemiological data on estimated sarcopenia shows wide variations due to the different diagnostic criteria, measuring methods for muscle mass, differences in the cut‐off points applied, and heterogeneity of study populations [[12], [13], [14]]. A single diagnostic criterion was not established until a consensus in 2019 and varied from Europe to Asia [15]. Therefore, the findings from studies regarding disability incidence among older adults whose sarcopenia was determined using the European criteria might not apply to China [14]. In addition, limited attention was focused on the IADLs, and the association between sarcopenia and IADLs disability was controversial [8,16]. The new operational definition of sarcopenia needs to show its relationship to ADLs and IADLs disability.

We used the nationally representative data from the China Health and Retirement Longitudinal Study (CHARLS) in conducting a cross‐sectional and longitudinal analysis to explore the association between sarcopenia and functional disability in the older Chinese adults, aiming to provide objective scientific evidence on prevention strategies of disability.

## 2 Methods

### 2.1 Study population

The CHARLS is a nationally representative longitudinal survey of participants over age 45 in China, intended to serve the needs of scientific and policy research on aging-related issues. More detailed study design could be found elsewhere [[17], [18], [19]], and this was a secondary analysis of the CHARLS. In brief, the baseline survey for CHARLS was established in 2011. Samples were chosen through multistage stratified probability-proportionate-to-size sampling in 10,257 households from 450 communities and 150 counties or districts in 28 provinces. The participants were interviewed by a structured questionnaire face-to-face in their homes through computer-assisted personal interviewing technology. All participants underwent the assessments of the social, economic, and health circumstances by standardized questionnaires and were followed up biennially. To date, CHARLS has released four waves’ data which can be downloaded at the CHARLS home page at http://charls.pku.edu.cn/en. We retrospectively analyzed data from the CHARLS 2015y and 2018y to provide more evidence on the association between sarcopenia and functional disability among older adults.

The sample size in 2015y was 21 097. We adopted 60 years as the cutoff for old age based on the definition of “old age” in contemporary Chinese society [18,20] and included participants aged at least 60. We excluded participants due to (1) missing data on sarcopenia status in 2015y; (2) missing data on ADLs and IADLs status in 2015y; (3) no information about age and sex in 2015y. For longitudinal analysis, we also excluded the participants with functional disability in 2015y and missing information on ADLs and IADLs status in 2018y.

### 2.2 Assessment of sarcopenia status

Sarcopenia was assessed according to the Asian Working Group for Sarcopenia (AWGS) 2019 algorithm and depended on three components: muscle strength, appendicular skeletal muscle mass (ASM), and physical performance [6]. Possible sarcopenia was defined as low muscle strength with or without low physical performance. Sarcopenia was defined as low muscle mass plus low muscle strength or combined low physical performance. Total sarcopenia refers to those people with either possible sarcopenia or sarcopenia. While participants without any low muscle strength, low muscle mass, and low physical performance were defined as having no sarcopenia.

Handgrip strength (unit: kg) was measured in the dominant and non-dominant hands using a Yuejian^TM^ WL-1000 dynamometer. Participants were instructed to squeeze the dynamometer as hard as possible, and the average of the two maximum readings for each hand was used in the analysis [17]. The cut-off points for low grip strength for males and females were <28 and <18 kg, respectively [6].

A validated anthropometric equation estimated the ASM in Chinese residents [21]:

ASM=0.193×weight+0.107×height-4.157×sex-0.037×age-2.631

Where the weight (unit: kg) was measured by Omron™ HN-286 scale, the height (unit: cm) was measured by Seca™ 213 stadiometer, and the sex “male” and “female” were coded into 1 and 2, respectively. The cut-off for defining low muscle mass was based on the sex-specific lowest 20% of the height-adjusted muscle mass (ASM/height^2^) among the study population. Finally, the ASM/height^2^ values of <5.07 kg/m^2^ in females and <6.87 kg/m^2^ in males were considered low muscle mass.

The gait speed and the chair stand test were considered in terms of physical performance. According to the requirement, the number of seconds the respondent took to walk 2.5 meters at their normal pace was recorded by a stopwatch during the first and second trials (there and back), respectively. Gait speed was assessed by measuring the participants’ usual gait (unit: m/s) in a 2.5-meter course [17]. The 5-time chair stand test measured the time needed for the participants to rise continuously five times from the height of the 47-centimeter chair. The respondent was instructed to keep their arms folded across their chest, stand up straight and then sit down again at their fastest pace five times without stopping in between and without using their arms to push off [17]. The timing was initiated when the participant stood up for the first repetition and stopped when they sat down after completing the fifth repetition. According to AWGS 2019, the criteria for low physical performance are a 6-meter walk <1.0 m/s or a 5-time chair stand test ≥12 s [6].

### 2.3 Assessment of outcomes

The outcome of the study was functional disability, consisting of ADLs and IADLs. ADLs were the skills required to perform daily physical tasks, including dressing, bathing, feeding, transferring from bed to coach, using the toilet, and urinary and fecal continence [22]. IADLs were defined herein as the participants having some difficulty living independently: making housework, cooking, shopping, managing money, and taking medication [23]. Each ADLs and IADLs item were classified as “Do not have any difficulty,” “Have difficulty but can still do it,” “Have difficulty and need help,” and “Cannot do it.” A code of 0 indicates that the respondent did not have any difficulty with the activity, and a code of 1 means that the respondent had difficulty with or could not do the activity. The sum of the items of ADLs and IADLs was calculated respectively into a score, with ≥1 score equal to a disability and 0 score equal to no disability.

### 2.4 Potential confounders

We also considered sociodemographic characteristics and health‐related factors as potential confounders. Sociodemographic variables included age, sex, marital status (married or remarriage and others), education background (elementary school or below, middle school, and high or above), and residence (rural, urban). Health-related factors included smoking, drinking status (yes, no), physical activity (yes, no), history of falls (yes, no), number of self-reported comorbidities, including cancer, chronic lung diseases, heart disease, stroke, emotional and mental disorders, arthritis, dyslipidemia, hepatic disease, kidney disease, digestive system disease, asthma, memory‐related disease, hypertension, and hyperglycemia, was classified as 0, 1, 2, ≥3. Physical examinations included systolic blood pressure, diastolic blood pressure, height, and weight. Fasting blood glucose and low-density lipoprotein cholesterol were measured using standard laboratory methods at the Youanmen Center for Clinical Laboratory of Capital Medical University. The enzymatic colorimetric test was used for analyses of fasting blood glucose (Within-assay: 0.90%, Between-assay: 1.80%, and Detection limits: 2−450 mg/dL), and low-density lipoprotein cholesterol (Within-assay: 0.70%, Between-assay: 1.20%, and Detection limits: 3−400 mg/dL). Body mass index was calculated as the weight (unit: kg) divided by the square of height (unit: m). Depression was assessed by the 10-item Center for Epidemiologic Studies Depression Scale, with 30 of the total scores [24]. Cognition was measured from four dimensions, including orientation (today’s year, month, day, the day of the week, and the current season), memory (recall and delayed recall tests of memory of 10 words), computation (5 tests of serial subtractions of 7 from 100), and drawing (reproduce a picture of two overlapped pentagons). The total cognition score was defined as the sum of four dimensions, resulting in 31 points [25].

## 3 Statistical methods

Data were presented as means ± standard deviation (SD) or median and interquartile range for continuous variables and percentages for categorical variables. Logistic regression analysis was used to estimate the associations between total sarcopenia, possible sarcopenia, sarcopenia, and functional disability in the cross-sectional study, expressed in odds ratios (ORs) and 95% confidence intervals (CIs). We also used a logistic regression model to analyze the association between sarcopenia status and the incidence of functional disability based on longitudinal data from 2015 and 2018. Different combinations of covariates were used in five adjustment models. More specifically, model 1 included only sarcopenia status; model 2 additionally included age, sex, marital status, residence, and education background; model 3 additionally included smoking status, drinking status, physical activity, and body mass index; and model 4 additionally included a history of fall, depression score, and cognition score; and model 5 additionally included comorbidity, systolic blood pressure, diastolic blood pressure, fasting blood-glucose, and low-density lipoprotein cholesterol. Given variations in muscle mass thresholds by sex [21] and differences in health status between urban and rural older adults [26], we conducted stratified analyses by sex and residence. Interaction terms were employed in our regression models to assess whether the effect of sarcopenia on functional disability was modified by sex or residence. We provided the baseline characteristics of the study population for the cross-sectional and longitudinal studies, as well as those who were lost to follow-up (Supplemental Table 1–3). We also examined the association between each component of sarcopenia and functional disability. To validate our results for appendicular muscle mass, we utilized an alternative equation: ASM = −24.337 − 0.173 × age (y) + 30.00 × height (m) + 0.115 × weight (kg) [27]. The predictive power of the sarcopenia for functional disability was assessed using the receiver-operating characteristic curve analysis. All statistical analyses were conducted using STATA 17.0 software, and the significance level of statistical tests was 0.05.

## 4 Results

**Fig. 1:**
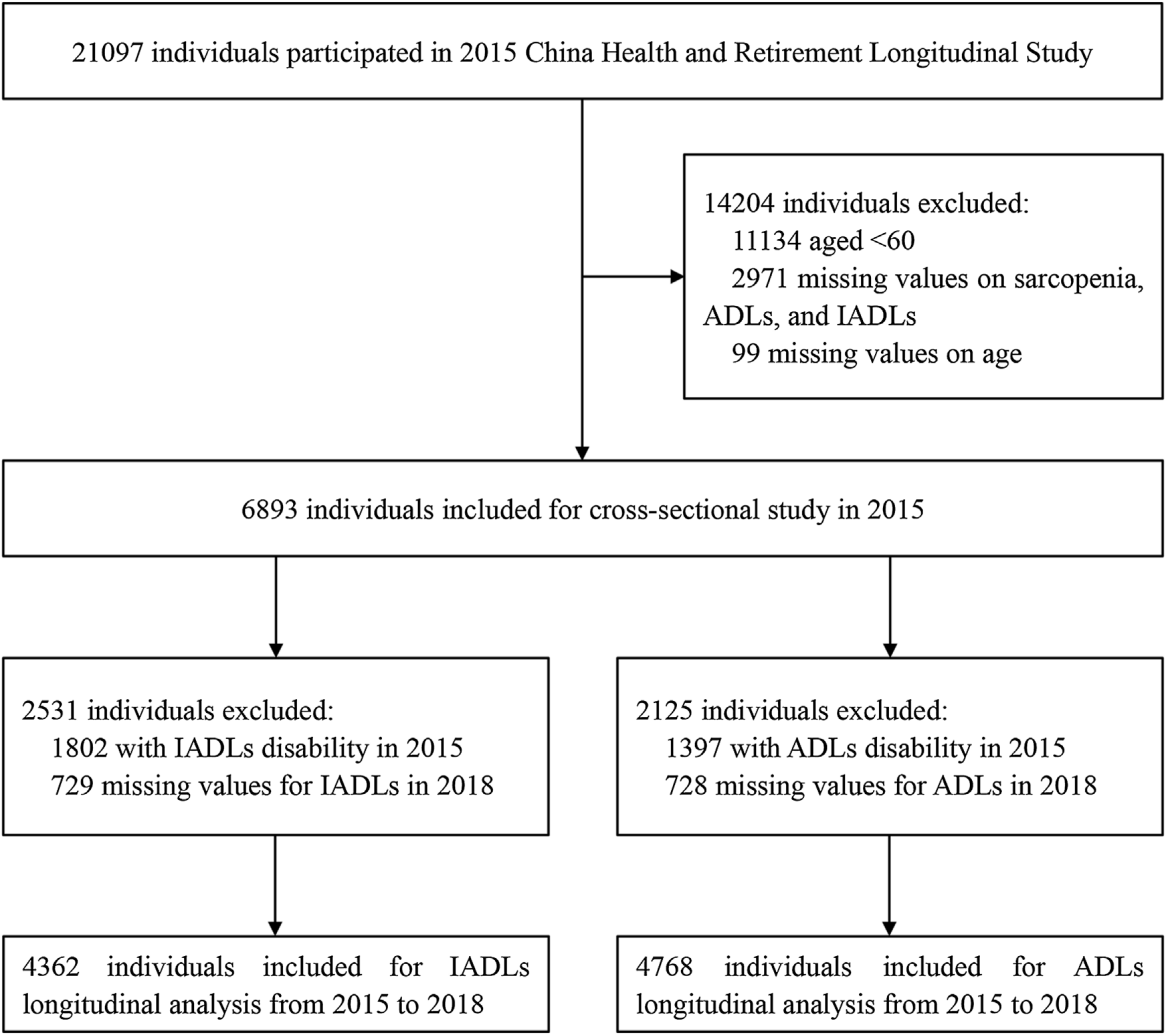
Flow diagram for participants included in the study.

In the current study, 21097 individuals participated in CHARLS 2015y, 11134 participants aged <60 were excluded, and 2971 participants were excluded for missing data on sarcopenia status, ADLs, and IADLs status in 2015y; 99 participants were excluded for no information about age. Finally, 6893 participants were included in the cross-sectional study. Then, further exclusion about functional disability in 2015y and missing information on ADLs and IADLs status in 2018y was done, and 4362 participants left for IADLs longitudinal analysis and 4768 participants left for ADLs longitudinal analysis. The detailed selection process was shown in Fig. 1.

The average age of the 6893 participants was 67.8 years (SD: 6.4 years), and 50.8% were male. Based on AWGS criteria, 2856(37.2%), 1856(26.9%) and 710(10.3%) participants were diagnosed with total sarcopenia, possible sarcopenia and sarcopenia, respectively. Compared with no sarcopenic, total sarcopenia individuals were more likely to be of more advanced age, female, rural residents, lower education, lower BMI, lower cognition score, more depression score, and to be with more comorbidities (Table 1). For the cross‑sectional study, the prevalence of ADLs disability in no-sarcopenia, total sarcopenia, possible sarcopenia, and sarcopenia groups was 16.7%, 33.4%, 34.1%, and 31.7%, respectively, and the prevalence of IADLs disability in no-sarcopenia, total sarcopenia, possible sarcopenia, and sarcopenia groups was 22.1%, 42.1%, 40.0%, and 48.3%, respectively (Supplemental Fig. 1). After adjustment for potential confounders at baseline, total sarcopenia (OR: 1.89, 95%CI: 1.66−2.15), possible sarcopenia (OR: 1.88, 95%CI: 1.64−2.16) and sarcopenia (OR: 1.91, 95%CI: 1.54−2.39) were significantly associated with ADLs disability compared to individuals without sarcopenia. Similarly, total sarcopenia (OR: 1.80, 95%CI: 1.60−2.03), possible sarcopenia (OR: 1.74, 95%CI: 1.53−1.99) and sarcopenia (OR: 2.00, 95%CI: 1.64−2.44) were still significantly associated with IADLs disability compared to individuals without sarcopenia (Table 2).

**Table 1:** Baseline characteristics of the study population in the cross-sectional study.

	No sarcopenia	With sarcopenia		
		Total sarcopenia	Possible sarcopenia	Sarcopenia
Participants	4327	2566	1856	710
Age, year	66.3 ± 5.4	70.4 ± 7.1	69.0 ± 6.5	74.0 ± 7.3
BMI, kg/m^2^	23.5 ± 3.7	23.1 ± 4.2	24.7 ± 3.7	18.9 ± 1.7
Depression, score	6.0 (3.0, 10.0)	8.0 (4.0, 13.0)	8.0 (4.0, 13.0)	8.0 (4.0, 14.0)
Cognition, score	15.1 ± 4.8	13.2 ± 5.0	13.5 ± 5.0	12.2 ± 5.1
Grip strength, kg	31.5 ± 8.1	22.6 ± 8.1	23.5 ± 8.3	20.1 ± 7.2
Gait speed, m/s	0.8 (0.7,1.0)	0.7 (0.5, 0.8)	0.7 (0.6−0.8)	0.6 (0.5−0.8)
5-time chair stand test, s	8.5 ± 1.8	13.5 ± 4.6	13.5 ± 4.5	13.2 ± 4.8
ASM/Ht^2^, kg/m^2^	6.7 ± 1.1	6.5 ± 1.2	6.8 ± 1.1	5.5 ± 1.0
SBP, mmHg	130.1 ± 19.9	132.3 ± 21.6	133.7 ± 20.8	128.4 ± 23.0
DBP, mmHg	74.3 ± 11.0	73.7 ± 11.4	75.0 ± 11.3	70.4 ± 11.3
FBG, mg/dL	104.0 ± 31.2	105.2 ± 35.0	107.1 ± 36.0	100.1 ± 31.8
LDL-C, mg/dL	103.9 ± 28.8	103.3 ± 28.4	104.6 ± 28.0	99.9 ± 29.1
Male	2296 (53.1)	1209 (47.1)	873 (47.0)	336 (47.3)
Married or remarriage	3659 (84.6)	1892 (73.7)	1416 (76.3)	476 (67.0)
Residence				
Urban	1739 (40.2)	813 (31.7)	666 (35.9)	147 (20.7)
Rural	2588 (59.8)	1753 (68.3)	1190 (64.1)	563 (79.3)
Education background				
Elementary or below	3295 (76.1)	2199 (85.7)	1549 (83.5)	650 (91.5)
Middle school	669 (15.5)	262 (10.2)	220 (11.9)	42 (5.9)
High or above	363 (8.4)	105 (4.1)	87 (4.7)	18 (2.5)
Smoking	1300 (30.0)	676 (26.3)	466 (25.1)	210 (29.6)
Drinking	1590 (36.7)	714 (27.8)	520 (28.0)	194 (27.3)
Physical activity	3874 (89.5)	2186 (85.2)	1595 (85.9)	591 (83.2)
Fall	752 (17.4)	574 (22.4)	420 (22.6)	154 (21.7)
Comorbidity				
0	1573 (36.4)	787 (30.7)	531 (28.6)	256 (36.1)
1	1333 (30.8)	757 (29.5)	550 (29.6)	207 (29.2)
2	853 (19.7)	562 (21.9)	406 (21.9)	156 (22.0)
≥3	568 (13.1)	460 (17.9)	369 (19.9)	91 (12.8)

Data is n (%) or mean ± SD or median (*P*_25_, *P*_75_).

Abbreviations: BMI, body mass index; SBP, systolic blood pressure; DBP, diastolic blood pressure; FBG, fasting blood-glucose; LDL-C, low-density lipoprotein cholesterol.

**Table 2:** Cross-sectional association of sarcopenia status with functional disability, 2015 y.

	No sarcopenia (n = 4327)	With sarcopenia		
		Total sarcopenia (n = 2566)	Possible sarcopenia (n = 1856)	Sarcopenia (n = 710)
ADLs disability				
Model 1	Reference	2.51 (2.24−2.81)	2.58 (2.28−2.93)	2.32 (1.94−2.77)
Model 2	Reference	2.22 (1.97−2.51)	2.36 (2.07−2.68)	1.81 (1.49−2.20)
Model 3	Reference	2.21 (1.96−2.50)	2.23 (1.95−2.54)	2.16 (1.75−2.65)
Model 4	Reference	1.93 (1.70−2.20)	1.93 (1.68−2.21)	1.95 (1.57−2.43)
Model 5	Reference	1.89 (1.66−2.15)	1.88 (1.64−2.16)	1.91 (1.54−2.39)
IADLs disability				
Model 1	Reference	2.56 (2.31−2.85)	2.32 (2.07−2.61)	3.30 (2.81−3.89)
Model 2	Reference	2.09 (1.86−2.34)	2.03 (1.80−2.30)	2.28 (1.90−2.73)
Model 3	Reference	2.06 (1.84−2.31)	2.01 (1.78−2.28)	2.21 (1.82−2.68)
Model 4	Reference	1.82 (1.62−2.06)	1.76 (1.55−2.01)	2.02 (1.66−2.47)
Model 5	Reference	1.80 (1.60−2.03)	1.74 (1.53−1.99)	2.00 (1.64−2.44)

Abbreviations: ADLs, basic activities of daily living; and IADLs, instrumental activities of daily living.

Model 1: No adjustment. Model 2: Adjusted for age, sex, marital status, residence status, and education background. Model 3: Included covariates in model 2 and smoking status, drinking status, physical activity, and body mass index. Model 4: Included covariates in model 3 and history of fall, depression score, and cognition score. Model 5: Included covariates in model 4 and comorbidity, systolic blood pressure, diastolic blood pressure, fasting blood-glucose, and low-density lipoprotein cholesterol.

During three years of follow-up, 786 participants (16.5%) developed new‐onset ADLs disability, and 980 participants (22.5%) developed new‐onset IADLs disability. In no‐sarcopenia, total sarcopenia, possible sarcopenia, and sarcopenia groups, the incidence of ADLs disability was 12.8%, 24.5%, 24.1%, and 25.8%, respectively, and the incidence of IADLs disability was17.6%, 33.1%, 31.9%, and 41.5%, respectively (Supplemental Fig. 2). After adjustment for potential confounders, compared with the no‐sarcopenia, the risk of ADLs disability for participants with total sarcopenia, possible sarcopenia, and sarcopenia were 1.58(1.34−1.88), 1.62(1.34−1.95), and 1.58(1.18−2.11). While, the risk of IADLs disability for participants with total sarcopenia, possible sarcopenia was 1.71(1.45−2.00), 1.68(1.41−2.00), and 1.87(1.40−2.51), respectively (Table 3).

**Table 3:** Longitudinal association of sarcopenia status with functional disability, 2015–2018 y^a^.

	No sarcopenia	With sarcopenia		
		Total sarcopenia	Possible sarcopenia	Sarcopenia
ADLs disability				
Participants	3279	1489	1097	392
Model 1	Reference	2.20 (1.50−2.10)	2.15 (1.81−2.56)	2.36 (1.84−3.02)
Model 2	Reference	1.78 (1.50−2.10)	1.85 (1.55−2.21)	1.55 (1.18−2.03)
Model 3	Reference	1.76 (1.49−2.08)	1.79 (1.50−2.15)	1.65 (1.24−2.20)
Model 4	Reference	1.62 (1.37−1.92)	1.66 (1.38−1.99)	1.58 (1.18−2.11)
Model 5	Reference	1.58 (1.34−1.88)	1.62 (1.34−1.95)	1.58 (1.18−2.11)
IADLs disability				
Participants	3065	1297	998	299
Model 1	Reference	2.43 (2.09−2.81)	2.20 (1.87−2.58)	3.33 (2.60−4.27)
Model 2	Reference	1.85 (1.57−2.16)	1.83 (1.54−2.17)	1.92 (1.46−2.51)
Model 3	Reference	1.83 (1.56−2.15)	1.81 (1.53−2.15)	1.91 (1.44−2.55)
Model 4	Reference	1.73 (1.47−2.03)	1.70 (1.42−2.02)	1.88 (1.41−2.50)
Model 5	Reference	1.71 (1.45−2.00)	1.68 (1.41−2.00)	1.87 (1.40−2.51)

Abbreviations: ADLs, basic activities of daily living; and IADLs, instrumental activities of daily living.

Mean follow-up period = 3.0 years.

Model 1: No adjustment. Model 2: Adjusted for age, sex, marital status, residence status, and education background. Model 3: Included covariates in model 2 and smoking status, drinking status, physical activity, and body mass index. Model 4: Included covariates in model 3 and history of fall, depression score, and cognition score. Model 5: Included covariates in model 4 and comorbidity, systolic blood pressure, diastolic blood pressure, fasting blood-glucose, and low-density lipoprotein cholesterol.

a Participants with the outcomes at baseline were excluded.

**Fig. 2:**
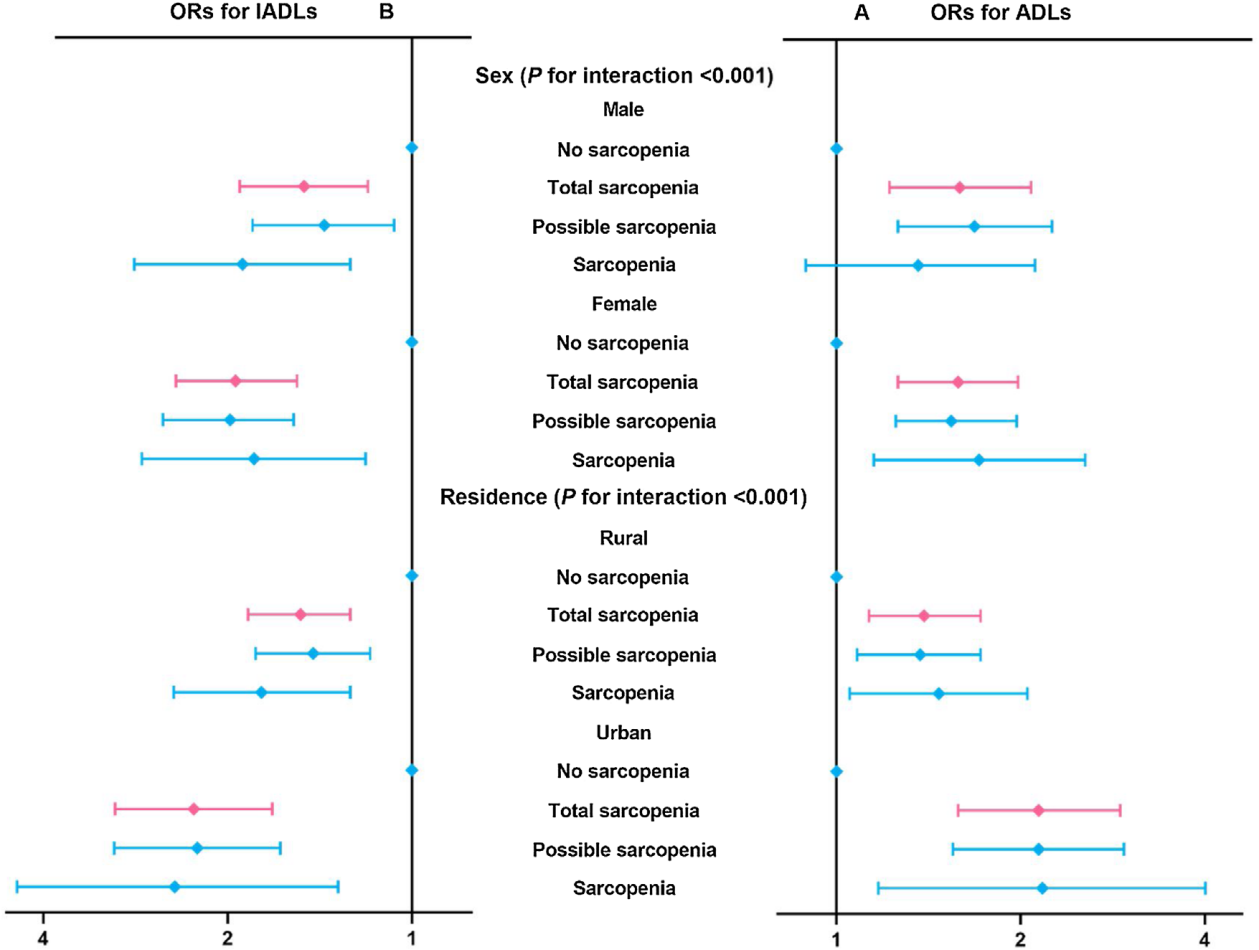
Longitudinal association of baseline sarcopenia status with ADLs and IADLs disability stratified by sex and residence status, 2015–2018 y. Abbreviations: OR, odds ratio; CI, confidence interval; ADLs, basic activities of daily living; and IADLs, instrumental activities of daily living. Mean follow-up period = 3.0 years. Adjusted for age, sex, marital status, residence status, education background, smoking status, drinking status, physical activity, body mass index, history of fall, depression score, cognition score, comorbidity, systolic blood pressure, diastolic blood pressure, fasting blood-glucose, and low-density lipoprotein cholesterol.

After adjustment for variables in model 5, stratified analyses showed significant differences in the effect of sarcopenia on functional disability by sex and residence (both *P* for interaction <0.001). For ADLs disability, total sarcopenia (OR: 1.58, 95%CI: 1.26–1.98), possible sarcopenia (OR: 1.54, 95%CI: 1.25–1.97) and sarcopenia (OR: 1.71, 95%CI: 1.15–2.55) were significantly associated with ADLs disability in females, but just total sarcopenia (OR: 1.59, 95%CI: 1.22–2.08) and possible sarcopenia (OR: 1.68, 95%CI: 1.26–2.25) showed an increased risk of ADLs disability in males. The ORs (95%CI) of total sarcopenia, possible sarcopenia, and sarcopenia in urban were 2.14(1.58–2.91), 2.14(1.55–2.95), and 2.17(1.17–4.01), respectively, and in rural were 1.39(1.13–1.72), 1.37(1.08–1.72) and 1.47(1.05–2.05), respectively (Fig. 2). For IADLs disability, the ORs (95%CI) of total sarcopenia, possible sarcopenia, and sarcopenia in males were 1.50(1.18–1.91), 1.39(1.07–1.82) and 1.89(1.26–2.84), respectively, and in females were 1.94(1.54–2.43), 1.98(1.56–2.52) and 1.81(1.19–2.76), respectively; in urban were 2.27(1.69–3.05), 2.24(1.64–3.06) and 2.44(1.32–4.51), respectively; and in rural were 1.52(1.26–1.85), 1.45(1.17–1.80) and 1.76(1.26–2.45), respectively (Fig. 2). The results in the cross-sectional study stratified by sex and residence were similar (Supplemental Table 4–5). For each component of sarcopenia, most of the results did not reach statistical significance (Supplemental Table 6–7). Furthermore, our study was strengthened by the inclusion of a new equation, and this new calculation did not alter our results or conclusions (Supplemental Table 8–9). The values of area under curve for ADLs and IADLs in 2015 were 0.76 and 0.75, but for incident ADLs and IADLs in 2015–2018 were 0.71 and 0.73 (Supplemental Table 10).

## 5 Discussion

To our knowledge, this is the first nationally representative study to examine the association between sarcopenia status and functional disability in older Chinese adults. In our study, we found that older adults with any form of sarcopenia were also associated with an increased risk of developing new-onset ADLs and IADLs disability. Older adults in urban were at higher risk of sarcopenia-related ADLs and IADLs disability than those in rural. Interestingly, in the sex-stratified analysis, possible sarcopenia demonstrated its stronger association. The possible sarcopenia had a much higher risk of ADLs disability in males, while in females, the possible sarcopenia had a higher risk of IADLs disability.

Previous studies suggested that sarcopenia was associated with a higher risk of functional disability irrespective of differences in the measurement and criteria of sarcopenia [11,14]. A cross-sectional study conducted among the high-income urban oldest old (≥80 years old) showed that the prevalence of sarcopenia was 26.6%, and the odds of disability in ADLs was 1.94-fold greater in the oldest old with sarcopenia than that without [11]. Given that our study population was relatively young and the region covered both rural and urban areas, the result of our cross-sectional study indicated that participants with total sarcopenia had 89% higher odds of reporting difficulty in at least 1 ADL than people without sarcopenia. In a nationwide study in Japan, the older with sarcopenia were classified in different way as having probable sarcopenia (poor handgrip strength), sarcopenia (poor handgrip strength and low muscle mass), or severe sarcopenia (sarcopenia with low walking speed). Over 49-month follow-up, compared with no-sarcopenia, although the risk of disability was a 2.18-fold increase in participants with severe sarcopenia, there was no statistical difference in disability risk among participants with sarcopenia [14]. While in our longitudinal study, older people with any form of sarcopenia were at higher risk of incident ADLs and IADLs disability, which may be caused by different threshold values and definitions of sarcopenia [12].

In addition to sarcopenia, the current study also demonstrated interesting association between possible sarcopenia and functional disability. A prospective study in Japan showed that possible sarcopenia, which does not belong to either the reference or the sarcopenia group, was not associated with significantly increased risk of incident disability [28]. However, we provided the evidence that the risks of ADLs and IADLs disability were significantly increased in participants with possible sarcopenia. Possible sarcopenia was defined as low muscle strength with or without low physical performance [6]. Higher muscle strength could provide individuals with a protective reserve against the development of ADLs and IADLs disability [29], and a previous study reported that the prevalence of ADLs and IADLs disability increases with muscle strength shown to decrease [30]. The gait speed mediated the negative effect of sarcopenia on functional dependence [31], and the chair stand test was also significantly associated with worsening functional disability [32]. Our findings suggested that maintaining adequate muscle strength and/or physical performance could contribute to preventing functional disability for older adults. Meanwhile, early discovery, diagnosis, and treatment of possible sarcopenia are necessary to elevate the quality of life for older adults.

Limited studies evaluated the association between sarcopenia and functional disability stratified by sex and residence status, and no consistent conclusions have been reached. A study conducted in Japan reported a 1.6-fold increase in the risk of disability for males and a 1.7-fold increase for females with sarcopenia compared to those without, while pre-sarcopenia showed no significant association with disability in either gender [28]. In the Concord Health and Ageing in Men Project, different types of sarcopenia were associated with an increased risk of disability in males, based on criteria from the Foundation for the National Institutes of Health [13]. While in our study, we supported that sarcopenia was associated with functional disability in both older males and females, with males in the total sarcopenia group exhibiting a higher risk of ADLs disability compared to those without sarcopenia, while females were more susceptible to IADLs disability. However, upon further classification of sarcopenia status, our research showed that possible sarcopenia was significantly associated with a higher risk of ADLs disability in males and IADLs disability in females compared to sarcopenia. Possible reasons include the fact that sarcopenia was primarily defined by low muscle mass, while possible sarcopenia was characterized by low grip strength or reduced physical performance. Consequently, different components of sarcopenia may be more closely related to specific functional activities, and different genders may exhibit varying degrees of impairment when performing these activities. Furthermore, our findings indicated that older adults with any form of sarcopenia faced an elevated risk of ADLs and IADLs disability, both in urban and rural residence, with stronger association observed in urban older adults. Urban environments often present different challenges and lifestyle factors that can exacerbate the effects of sarcopenia, such as reduced access to physical activity opportunities or increased sedentary behaviors [33]. It's important to note that rural areas may also have unique factors, such as terrain and agricultural labor, and most Chinese rural adults still remain in farming habits and perform more manual labor that can influence the relationship between sarcopenia and disability [34,35]. Therefore, different strategies should be taken varying by sex and place of residence to prevent sarcopenia.

There are several limitations in the current study. Firstly, we utilized an anthropometric equation, previously validated in Chinese populations [36], to estimate muscle mass, deviating from the AWGS recommendation of using DEXA [15]. While anthropometric measures provide a rough estimate for assessing muscle mass, DEXA poses cost and X-ray exposure concerns. Secondly, the AWGS recommended using a 6-meter usual gait speed for measuring physical performance rather than 2.5-m in CHARLS, but a systematic review indicated that distance did not influence the gait speed [37]. Thirdly, due to the specific distribution of our sample, which comprised 37.02% urban and 62.98% rural individuals, the findings should be interpreted within the context of the sampled population and may not be fully representative of broader rural and urban strata. Fourthly, the longitudinal study was not specially designed for the association between sarcopenia and functional disability. Although we adjusted potential confounders based on prior knowledge, some extra confounders remain, such as dwelling environment and nutrition status, which could lead to an inaccurate interpretation of the study results. Additionally, we excluded participants lost to follow-up, considering that this group had older age, worse performance, and lower ASM compared to the follow-up cohort. This may lead to an underestimation of the study results.

In conclusion, both possible sarcopenia and sarcopenia were associated with a higher risk of ADLs and IADLs disability among older adults in China. Our study provided new evidence supporting the longitudinal association between possible sarcopenia and ADLs and IADLs disability and highlighted the importance of sex and residential differences in these associations. Interventions aimed at preventing and treating possible sarcopenia and sarcopenia may be beneficial in reducing the progression of functional disability and promoting healthy aging among the older population.

## Authors’ contribution

Hui Zhou: conceptualization, writing and editing the original draft. Xiong Ding: methodology, data interpretation, validation, reviewing and editing the original draft. Meijie Luo: methodology, validation, and formal analysis. All authors agreed to be fully accountable for ensuring the integrity and accuracy of the work, and read and approved the final manuscript.

## Funding

None.

## Ethical guidelines statement

The CHARLS was approved by the Biomedical Ethics Committee of Peking University, and all participants were required to sign informed consent. All procedures performed in studies followed the Helsinki Declaration.

## Conflict of interests

None.
